# The complete mitochondrial genome of the striped hamster (*Cricetulus barabesis*) China and its phylogenetic analysis

**DOI:** 10.1080/23802359.2019.1641440

**Published:** 2019-07-16

**Authors:** Liu Zhu, Qin Ke-Song, Li Bo-Qi, Yu Cheng-Wen, Cai He, Zhang Jun-Sheng, Li Jin-Xun, Wang Zhu

**Affiliations:** College of Life Science and Technology, Mudanjiang Normal University, Mudanjiang, P.R. China

**Keywords:** Control region, mitogenome, phylogenetic trees, *Cricetulus barabesis*

## Abstract

The complete mitogenome sequence of the striped hamster was determined using long PCR. The genome was 16,282 bp in length and contained 13 protein-coding genes, two ribosomal RNA genes, 22 transfer RNA genes, one origin of L strand replication, and one control region. The overall base composition of the heavy strand is A (33.7%), C (22.8%), T (30.5%), and G (13.0%). The base compositions present clearly the A–T skew, which is most obviously in the control region and protein-coding genes. Mitochondrial genome analyses based on MP, ML, NJ, and Bayesian analyses yielded identical phylogenetic trees. Results of phylogenetic analysis showed that *Cricetulus* had close relationship with *Meriones*. This study verifies the evolutionary status of the striped hamster in *Cricetulus* at the molecular level. The mitochondrial genome would be a significant supplement for the striped hamster genetic background. Results of phylogenetic analysis showed that the striped hamster had close relationship with *C. griseus* in *Cricetulus.*

In this paper, the complete mitochondrial genome of the striped hamster was sequenced for the first time on ABI 3730XL using a primer walking strategy and the long and accurate PCR, with five pairs of long PCR primers and with 14 pairs of sub-PCR primers. A muscle sample was obtained from a female the striped hamster captured from from Hulun Lake regions of Daxinganling Mountains in Inner Mongolia Province, China (48°37′20″N, 117°53′17″E). The specimen is stored in Animal and Plant Herbarium of Mudanjiang Normal University. The voucher number is NM2016104.

The mitochondrial genome is a circular double-stranded DNA sequence that is 16,282 bp long including 13 protein-coding genes, two rRNA genes, 22 tRNA genes, one origin of L strand replication, and one control region. The accurate annotated mitochondrial genome sequence was submitted to GenBank with accession number MN056361. The arrangement of the multiple genes is in line with other Cricetidae species (Triant and DeWoody 2006; Fan et al. 2011; Hao et al. 2011; Bendová et al. 2016; Chen et al. 2016; Cong et al. 2016; Kang et al. 2016; Luo and Liao 2016; Park et al. 2017) and most mammals (Meganathan et al. 2012; Yoon et al. 2013; Xu et al. 2012, 2013; Liu et al. 2017a, 2017b, 2017c, 2018, 2019a, 2019b; Jin et al. 2017).

The control region of the striped hamster mitochondrial genome was located between the tRNA-Pro and tRNA-Phe genes, and contains only promoters and regulatory sequences for replication and transcription, but no structural genes. Three domains were defined in the striped hamster mitochondrial genome control region (Zhang et al. 2009): the extended termination-associated sequence (ETAS) domain, the central conserved domain (CD) and the conserved sequence block (CSB) domain.

The total length of the protein-coding gene sequences was 11,389 bp. Most protein-coding genes initiate with ATG except for ND1, ND2, ND3 and ND5, which began with ATA, ATT, or GTG. Nine protein-coding genes terminated with TAA. The incomplete stop codons (T––) were used in COX3, and ND4. A strong bias against A at the third codon position was observed in the protein-coding genes. The frequencies of CTA (Leu), ATT (Ile), TTA (Leu) and ATA (Met) were higher than those of other codons. The length of tRNA genes varied from 59 to 75 bp. Twenty-one of them could be folded into the typical cloverleaf secondary structure except the tRNA-Ser (AGY), whose complete dihydrouridine arm was lacking.

Most the striped hamster mitochondrial genes were encoded on the H strand, except for the ND6 gene and eight tRNA genes, which were encoded on the L strand. Some reading frame intervals and overlaps were found. One of the most typical was between ATP8 and ATP6. The L-strand replication origin (OL) was located within the WANCY region containing five tRNA genes (tRNATrp, tRNA-Ala, tRNA-Asn, tRNA-Cys, tRNA-Tyr). This region was 31 bp long and had the potential to fold into a stable stem-loop secondary structure. The total base composition of the striped hamster mitochondrial genome was A (33.7%), C (22.8%), T (30.5%), and G (13.0%). The base compositions clearly present the A-T skew, which was most obviously in the control region and proteincoding genes.

In order to explore the evolution of Cricetidae species which include 28 genera, especially the evolution of genus *Cricetulus*, here, we investigate the molecular phylogenetics of Chinese the striped hamster using complete mitochondrial genome sequence of 55 species. All sequences generated in this study have been deposited in the GenBank (Figure 1).

**Figure 1.**
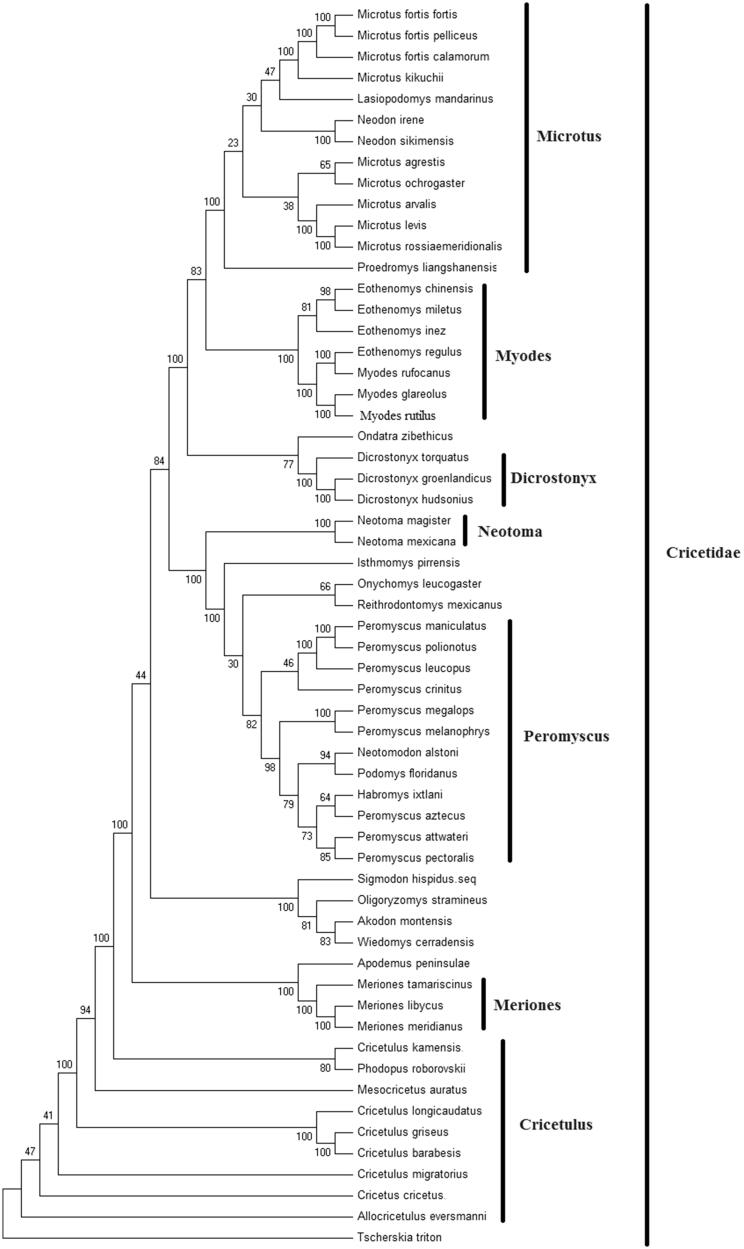
Phylogenetic tree generated using the Maximum Parsimony method based on complete mitochondrial genomes. *Akodon montensis* (KF769456), *Allocricetulus eversmanni* (KP231506), *Cricetulus barabesis* (MN056361)*, C. kamensis* (KJ680375), *C. griseus* (DQ390542), *C. migratorius* (KT918407), *C. longicaudatus* (KM067270), *Cricetus cricetus* (MF405145), *Dicrostonyx torquatus* (KX066190), *D. groenlandicus* (KX712239), *D. hudsonius* (KX683880), *Eothenomys miletus* (KX014874), *E. chinensis* (FJ483847), *E. regulus* (JN629046), *E. inez* (KU200225), *Habromys ixtlani* (KY707304), *Isthmomys pirrensis* (KY707312), *Lasiopodomys mandarinus* (KF819832), *Meriones meridianus* (KR013227), *M. libycus* (KR013226), *M. tamariscinus* (KT834971), *Mesocricetus auratus* (EU660218), *Microtus rossiaemeridionalis* (DQ015676), *M. f. Pelliceus* (MK805519)*, M. f. calamorum* (JF261175)*, M. f. fortis* (JF261174), *M. levis* (NC 008064), *M. kikuchii* (AF348082), *M. ochrogaster* (KT166982), *M. arvalis* (MG948434), *M. agrestis* (MH152570), *Myodes glareolus* (KF918859), *M. rufocanus* (KT725595), *Neodon irene* (HQ416908), *N. sikimensis* (KU891252), *Neotoma mexicana* (KY707300), *N. magister* (MG182016), *Neotomodon alstoni* (KY707310), *Onychomys leucogaster* (KU168563), *Oligoryzomys stramineus* (MF696155), *Ondatra zibethicus* (KX377613), *Peromyscus maniculatus* (MH260579), *P. leucopus* (MH256659), *P. megalops* (KY707305), *P. crinitus* (KY707308), *P. melanophrys* (KY707303), *P. polionotus* (KY707301), *P. pectoralis* (KY707309), *P. aztecus* (KY707306), *P. attwateri* (KY707299), *Phodopus roborovskii* (KU885975), *Proedromys. liangshanensis* (FJ463038), *Podomys floridanus* (KY707302), *Reithrodontomys mexicanus* (KY707307), *Sigmodon hispidus* (KY707311), *Apodemus peninsulae* (JN546584), and *Wiedomys cerradensis* (KF769457). The out group is *Tscherskia triton* (EU031048).

Mitochondrial genome analyses based on MP, ML, NJ, and Bayesian analyses yielded identical phylogenetic trees, indicating a close phylogenetic affinity of species. The phylogram obtained from Maximum Parsimony method is shown in Figure 1. Results of phylogenetic analysis showed that *Cricetulus* had close relationship with *Meriones*. This study verifies the evolutionary status of the striped hamster in *Cricetulus* at the molecular level. The mitochondrial genome would be a significant supplement for the striped hamster genetic background. Results of phylogenetic analysis showed that the striped hamster had close relationship with *C. griseus* in *Cricetulus.*
